# Effectiveness and Safety of the Combination of the Traditional Chinese Medicine Prescription Jade Screen and Desloratadine in the Treatment of Chronic Urticaria: A Systematic Review and Meta-Analysis of Randomized Controlled Trials

**DOI:** 10.1155/2017/1390301

**Published:** 2017-11-12

**Authors:** Shao-fei Yuan, Jing-zhi Guan, Yong Hao, Jian-yuan Chai, Feng-li Gao, Su-qin Shi, Jian-xin Wang, Jin He, Ji-hai Shi

**Affiliations:** ^1^Pharmaceutical Department, The Second Affiliated Hospital of Baotou Medical College, No. 30 Hudemulin Road, Baotou, Inner Mongolia 014030, China; ^2^Pharmaceutical Department, Inner Mongolia International Mongolian Hospital, No. 83 Daxue East Road, Hohhot, Inner Mongolia 010065, China; ^3^Dermatological Department, The Second Affiliated Hospital of Baotou Medical College, No. 30 Hudemulin Road, Baotou, Inner Mongolia 014030, China; ^4^Department of Gastroenterology, The Second Affiliated Hospital of Baotou Medical College, No. 30 Hudemulin Road, Baotou, Inner Mongolia 014030, China; ^5^Human Anatomy and Tissue Embryology, Weifang Medical University, No. 7166 Baotong Street, Weicheng District, Weifang, Shandong 261021, China; ^6^Development Department, Inner Mongolia International Mongolian Hospital, No. 83 Daxue East Road, Hohhot, Inner Mongolia 010065, China

## Abstract

**Objective:**

The aim of this study is to systematically evaluate the clinical efficacy and safety of the traditional Chinese medicine prescription Jade Screen combined with desloratadine in the treatment of chronic urticaria.

**Methods:**

Two researchers independently conducted literature searches. The extracted data were analyzed using Rev Man 5.2.3 software. The established retrieval time range of the various databases was up to 15 March, 2017.

**Results:**

Sixteen randomized controlled trials were included in this study. The results of the meta-analysis showed that the total effective rate of using Jade Screen and desloratadine in combination to treat chronic urticaria was higher than that with desloratadine alone (*P* < 0.00001), while its recurrence rate (*P* < 0.00001) and symptom score (*P* = 0.006) were both significantly lower than the latter. The rate of adverse reaction in the combination group was lower than that when orally taking desloratadine alone (*P* = 0.74), and the serum level of total IgE in the combination group was lower than that when orally taking desloratadine alone (*P* = 0.82); however, the results of the rate of adverse reaction and the serum level of total IgE were insignificant.

**Conclusion:**

Using Jade Screen and desloratadine together to treat chronic urticaria gains a better clinical effect than using desloratadine alone.

## 1. Introduction

Urticaria is a common disease in dermatology, characterized by transient, localized, edematous wheals of various sizes on skin mucous membranes and accompanied by severe itching. If wheals occur every day or almost every day for more than six weeks, such condition is called chronic urticaria [1]. Approximately 0.5%~1.0% of the world population has chronic urticaria [2]. With the aggravation of urban pollution and the worsening of the environment, the incidence rate of urticaria is on the rise year by year. Since its occurrence shows no apparent pattern, the disease recurs easily, and the health index of the patients [3, 4] and their quality of life decline [5, 6]. The conventional treatment method involves the single use or combined use of antihistamine drugs [7]. However, these drugs can only relieve the symptoms of some patients, and the overall effect is not significant. The disease easily relapses after withdrawal [8]. Thus, there is an urgent need to seek new therapeutic strategies [2].

Urticaria is called “hidden rash” or “red and white wandering wind” in traditional Chinese medicine. As the “Treatise on Needling” in the Sù Wèn (“Plain Questions”) states, when shaoyin qi is excessive, skin numbness and hidden rash will occur, which is also recorded in *⟪*General Treatise on Causes and Manifestations of All Diseases*⟫*, *⟪*Invaluable Prescriptions for Ready Reference*⟫*, and *⟪*Complete Works of Diagnosis and Treatment for Surgical Diseases and other works*⟫*. Most modern doctors believe that rheumatism attacks the skin but decreased resistance and transportation dysfunction of the spleen and stomach are the keys to the disease [9]. It can be seen that strengthening the spleen and nourishing qi can not only expel the invasive wind-pathogen out of the body but also prevent it from spreading within the body. Traditional Chinese medicines strengthening the spleen, nourishing qi, dispelling wind, and arresting itching have the functions to improve the immune system and to eliminate pathogens, thereby controlling the cause and manifestation of chronic urticaria. Jade Screen is a classical prescription that comes from Danxi's Mastery of Medicine by Zhu Dan-xi, a great master of the Yuan Dynasty. As a basic Chinese medicine, this prescription has a good therapeutic effect. It has been listed into Chinese Pharmacopoeia [10]. Jade Screen formula consists of three traditional Chinese medicines, including* Astragalus membranaceus [Astragalus membranaceus *(Fisch.) Bunge*]*; Rhizoma Atractylodis Macrocephalae* (bighead atractylodes rhizome)*; and Radix Saposhnikoviae* [Saposhnikovia divaricata *(Turcz.) Schischk*]*.* Astragalus membranaceus* can nourish qi and strengthen the exterior to enhance the grain of the skin and the texture of the subcutaneous flesh and prevent it from evil invasion; Rhizoma Atractylodis Macrocephalae strengthens the middle warmer and invigorates the spleen to recover vital qi, and Radix Saposhnikoviae dispels wind and disperses evil to prevent external invasion and help cure various diseases. The compatibility of the three medicines has the functions of nourishing qi, strengthening the exterior, and arresting sweat. With sweet flavor and warm nature,* Astragalus membranaceus* tonifies qi and strengthens the exterior, while Rhizoma Atractylodis Macrocephalae invigorates the spleen and eliminates dampness. The combination of* Astragalus membranaceus* and Rhizoma Atractylodis Macrocephalae can greatly tonify lung and stomach qi, make the spleen and stomach healthy and vigorous, replenish the fleshy exterior, and prevent evil invasion, while Radix Saposhnikoviae relieves the exterior syndrome by dispersing wind-evil.

Among published clinical studies, some reports argue that Jade Screen combined with desloratadine can achieve a good therapeutic effect on chronic urticaria. To comprehensively assess the clinical efficacy and safety of this classical ancient prescription, we conducted this systematic review.

## 2. Methods and Analysis

### 2.1. Search Strategy

Records from the following databases in Chinese or English have been retrieved up to 15 March, 2017: Cochrane Library, PubMed, Embase, SinoMed, CNKI, VIP, and WanFang Data. The retrieval strategy was based on the combination of subject terms and free words. Chinese search terms were “Jade Screen”, “dilvleitading”, “xunmazhen”, etc.; English search terms were “Yu Ping Feng”, “Jade Screen”, “traditional Chinese medicine”, “Urticaria”, “Urticarias”, “desloratadine”, “Clarinex” and so on. Manual retrieval was conducted in *⟪*Lishizhen Medicine and Materia Medica Research*⟫*, *⟪*China Journal of Experimental Traditional Medical Formulae*⟫*, *⟪*Journal of Clinical Dermatology*⟫*, *⟪*the Chinese Journal of Dermatovenereology*⟫*, *⟪*Liaoning Journal of Traditional Chinese Medicine*⟫*, and relevant conference papers and proceedings after 2010 to improve the recall ratio.

### 2.2. Inclusion and Exclusion Criteria

#### 2.2.1. Type of Study

Jade Screen combined with desloratadine treatment of chronic urticaria in randomized controlled trials was included, including literature limited to the Chinese or English languages and regardless of whether the studies were blinded.

#### 2.2.2. Subject Investigated

① Patient age and sex were recorded. The patient's region is not limited, and the diagnosis of chronic urticaria is referred to as “urticaria”, *⟪*Chinese Medical Association clinical treatment guidelines. Skin disease and STD Volume*⟫*, *⟪*Practical Dermatology*⟫*, *⟪*Clinical dermatology*⟫*,” *⟪*Dermatology*⟫* [11–15]. ② Patients who recently did not take antihistamines and glucocorticoid drugs were included.

#### 2.2.3. Intervention

The test group is Jade Screen combined with desloratadine treatment; the control group is desloratadine alone treatment. In both groups, treatment time and dose were not limited.

#### 2.2.4. Outcome Indicators

① Total efficiency refers to EAACI/GA2LEN/EDF/WAO scoring criteria according to the severity of clinical symptoms using 4 scores; the total score is the sum of the individual scores. Symptom Score Reducing Index (SSRI) = (total score before treatment − total score after treatment)/total score before treatment × 100%. Cured means that SSRI is reduced by more than 90%; markedly means that SSRI is reduced by 60% to 89%; improved means that SSRI is reduced by 20% to 59%; invalid means that SSRI is reduced by 20% or less. The total effective rate (number of cured cases + significant cases)/the total number of patients × 100%; ② recurrence rate is occurrence of healing criteria in patients with follow-up after 4 weeks to 6 months; ③ adverse reaction rate is the main symptoms which are dry mouth, nausea, headache, dizziness, fatigue, and lethargy; ④ symptom score and ⑤ serum total IgE levels are outcome indicators.

#### 2.2.5. Exclusion Criteria

The exclusion criteria included the following: ① non-RCT literature; ② urticaria with other diseases; ③ patients with inconsistent baseline data; ④ sample content of less than 20 literature articles; ⑤ case reports, reviews, animal experiments, and articles with important information reported incomplete after contacting responders; or ⑥ treatment measures that did not meet the preselected standard settings.

#### 2.2.6. Data Extraction

The two researchers independently read the abstracts of the documents that were retrieved. According to the exclusion criteria, the articles with obvious nonconformity were excluded, and the articles remaining that met the inclusion criteria were obtained. Further, for the noncompliant documents, a form was used to indicate the reasons for the exclusion. Finally, the data of the included articles were extracted, including title, source, author, test design, research object, research method, intervention measures, outcome measurement and evaluation, statistical analysis, recurrence, adverse reaction report, and conclusion. The two researchers cross-checked their respective average results, and if there were differences, they decided whether or not the article was included by discussing or asking the third evaluator to evaluate the article.

### 2.3. Quality Evaluation

According to the bias risk assessment tool recommended by the Cochrane Review Handbook 5.2, there are six aspects: (1) random allocation method; (2) allocation scheme hidden; (3) whether the survey object, treatment plan implementer, or result measure was blinded; (4) the completeness of the resulting data; (5) selective reporting of findings; and (6) other bias sources. The two reviewers independently read the full text and conducted a quality evaluation and then cross-checked the results of the quality evaluation of the trials that were difficult to determine by discussion or by the third evaluator.

### 2.4. Sensitivity Analysis

To study the impact of individual studies on heterogeneity, when the heterogeneity was found, if the study was deleted and the heterogeneity was significantly reduced, the study was considered as the main source of heterogeneity; the study was then further read and evaluated.

### 2.5. Data Analysis

Meta-analysis was performed using RevMan5.3 software. In this study, the odds ratio (OR) and its 95% CI were used as the effect analysis statistic (MD) and its 95% CI analysis; if the same variables were not consistent, then the mean difference (WMD) and 95% CI analysis were used. The heterogeneity of the included studies was analyzed using the *χ*^2^ test (the test level was set to *α* = 0.1), and the size of the heterogeneity was determined quantitatively with *I*^2^ (*P* > 0.1, *I*^2^ > 50%), and the results were statistically heterogeneous (*P* ≤ 0.1, *I*^2^ > 50%), and the statistical heterogeneity was statistically significant (*P* > 0.1, *I*^2^ > 50%). After the exclusion of significant clinical heterogeneity, the random effects model was used for the meta-analysis. Significant clinical heterogeneity was analyzed by subgroup analysis or sensitivity analysis, or descriptive analysis was performed.

## 3. Results

### 3.1. Retrieval Results

**Figure 1:**
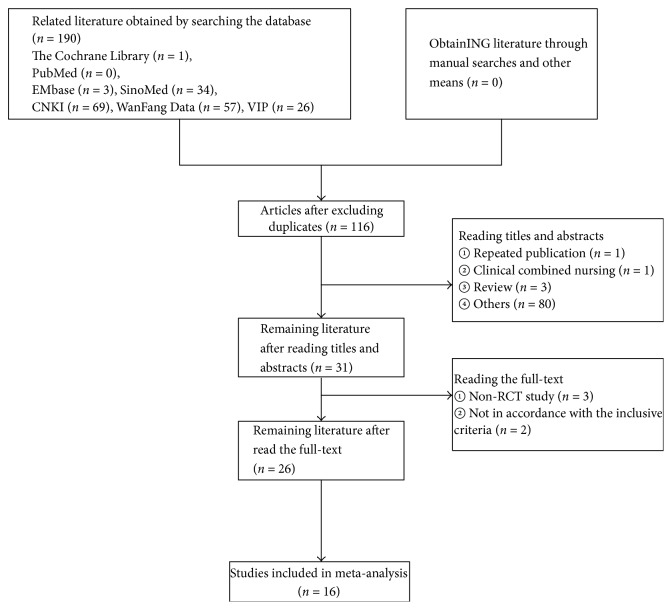
Flow diagram of the literature selection for this study.

A total of 190 relevant publications were found using the data collection method in accordance with the retrieval strategy, including 1 from the Cochrane Library, 0 from PubMed, 3 from Embase, 34 from SinoMed, 69 from CNKI, 57 from WanFang Data, and 26 from VIP. Among them, 116 papers were obtained after duplicate checking via literature management software, and 100 articles were excluded due to the failure to meet our inclusion criteria. Finally, 16 RCTs [16–31] involving 1763 patients were included.  Figure 1 shows the process and results of the literature screening.

### 3.2. Research Characteristics

There were 16 RCT studies that met our criteria and included in the study eventually [16–31]. These studies were published from 2008 to 2016 with a total of 1763 patients involved, including 901 in the experimental group and 862 in the control group. They were single center studies done in China. The age of the participants was from 12 to 75, and the course of treatment ranged from 3 weeks to 17 years, Table 1.

### 3.3. Summary of the Quality and Bias Risk of the Trials Included

**Figure 2:**
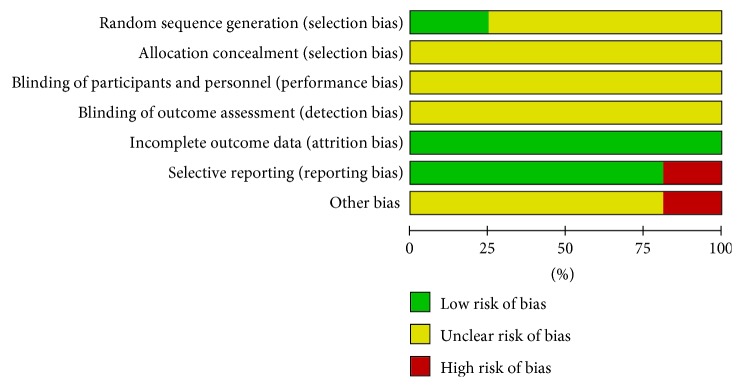
Risk of bias graph: review authors' judgments about each risk of bias item presented as percentages for all included studies.

**Figure 3:**
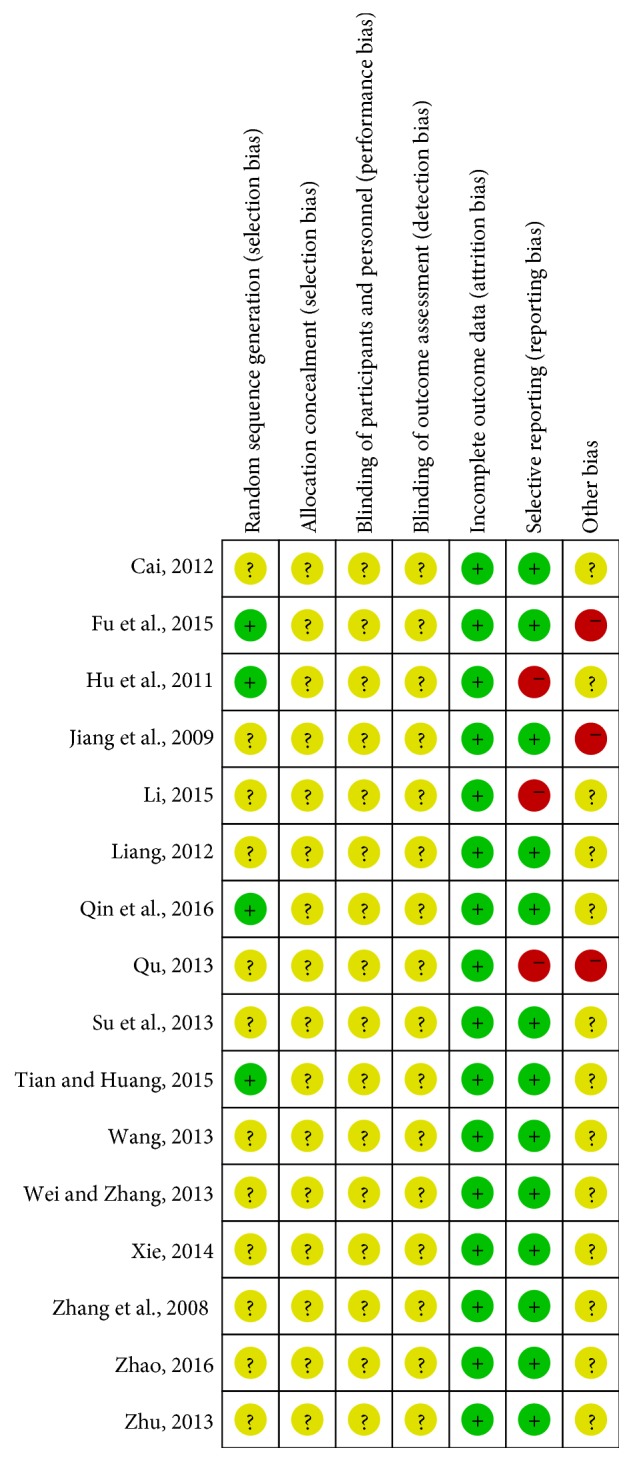
Risk of bias summary: review authors' judgments about each risk of bias item for each included study.

Among the 16 studies included, only 4 contained a random number table, and the rest failed to describe a random method, hidden group, blinding method, estimator's blinding method, loss to follow-up, and selection bias, Figures 2 and 3.

### 3.4. Outcome Measures

All 16 studies reported their total effective rate [16–31], but only some of them included other measurements; for instance, 8 reported their recurrence rates [19, 20, 22–24, 27, 30, 31], 7 described their rates of adverse reaction [19, 24, 25, 28–31], 7 mentioned their symptom scores, 7 reported their serum total IgE levels [27, 29], and 2 reported their serum total IgE level.

#### 3.4.1. Total Effective Rate

**Figure 4:**
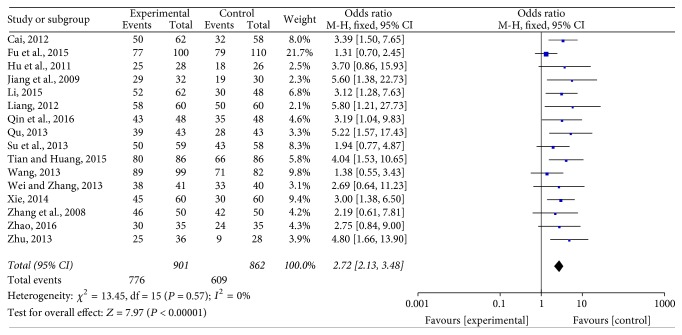
The meta-analysis forest map of the total effective rate of Jade Screen combined with desloratadine versus desloratadine alone for the treatment of chronic urticaria.

Among the 16 studies included [16–31], there was no heterogeneity found (*P* = 0.57, *I*^2^ = 0%). Using a fixed effects model, meta-analysis indicated that the overall response rate of Jade Screen combined with desloratadine for the treatment of chronic urticaria was significantly higher than that of the oral use of desloratadine alone [*n* = 16, OR = 2.72, 95% CI (2.13,3.48), *Z* = 7.97, *P* < 0.00001], Figure 4.

#### 3.4.2. Recurrence Rate

**Figure 5:**
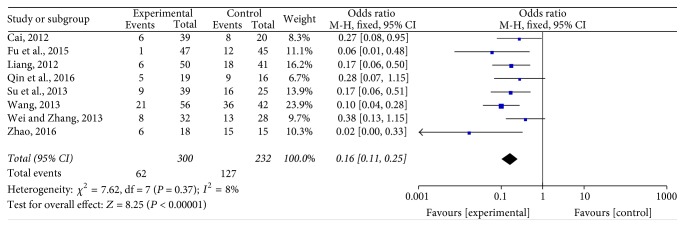
The meta-analysis forest map of the recurrence rate of Jade Screen combined with desloratadine versus desloratadine alone for the treatment of chronic urticaria.

This method [19, 20, 22–24, 27, 29, 31] was employed in 8 studies, involving 532 cases with 300 in the experimental group and 232 in the control group. Heterogeneity was still not found (*P* = 0.37, *I*^2^ = 8%). The fixed effects model revealed that the recurrence rate of Jade Screen combined with desloratadine for the treatment of chronic urticaria was significantly lower than that of the oral use of desloratadine alone [*n* = 8, OR = 0.16, 95% CI (0.11,0.25), *Z* = 8.25, *P* < 0.00001], Figure 5.

#### 3.4.3. Rate of Adverse Reaction

**Figure 6:**
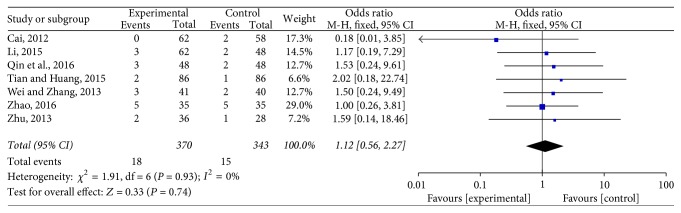
The meta-analysis forest map of the rate of adverse reaction of Jade Screen combined with desloratadine versus desloratadine alone for the treatment of chronic urticaria.

Seven studies adopted this method [19, 24, 25, 28–31], which involved 713 cases in total, including 370 in the experimental group and 343 in the control group. No heterogeneity was found (*P* = 0.93, *I*^2^ = 0%). Meta-analysis suggested that the rate of adverse reaction of Jade Screen combined with desloratadine for the treatment of chronic urticaria was lower than that of the oral use of desloratadine alone but failed to show a statistical significance [*n* = 7, OR = 1.12, 95% CI (0.56,2.27), *Z* = 0.33, *P* = 0.74 > 0.1], Figure 6.

#### 3.4.4. Symptom Score

**Figure 7:**
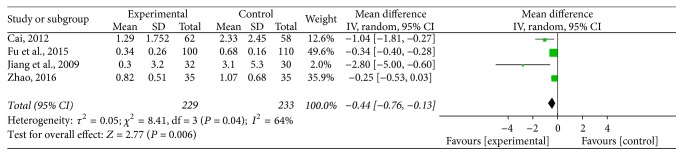
The meta-analysis forest map of the symptom scores of Jade Screen combined with desloratadine versus desloratadine alone for the treatment of chronic urticaria.

This method [17, 19, 27, 31] was employed in 4 studies involving 462 cases, including 229 in the experimental group and 233 in the control group. A significantly high heterogeneity was found (*P* = 0.04, *I*^2^ = 64%). The random effects model indicated that the symptom score of Jade Screen combined with desloratadine for the treatment of chronic urticarial was significantly lower than that of the oral use of desloratadine alone [*n* = 4, MD = −0.44, 95% CI (−0.76, −0.13), *Z* = 2.77, *P* = 0.006], Figure 7. For this measurement, we conducted sensitivity analysis, which showed that the heterogeneity was obviously decreased after deleting Jiang et al.'s research (2009). Thus, we believe that Jiang et al.'s research (2009) was the major heterogeneity source of the symptom score.

#### 3.4.5. Serum Total IgE Level

**Figure 8:**
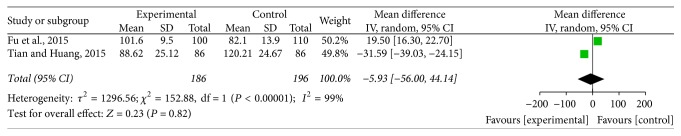
The meta-analysis forest map of the serum total IgE level of Jade Screen combined with desloratadine versus desloratadine alone for the treatment of chronic urticaria.

This method [27, 30] was employed in 2 studies involving 382 cases, including 186 in the experimental group and 196 in the control group. A significantly high heterogeneity was identified (*P* = 0.00001, *I*^2^ = 99%). The random effects model meta-analysis did not show a significant change between Jade Screen combined with the desloratadine group and the oral use of desloratadine alone [*n* = 2, MD = −5.93, 95% CI (−56.00, 44.14), *Z* = 0.23, *P* = 0.82 > 0.1], Figure 8.

### 3.5. Publication Bias

**Figure 9:**
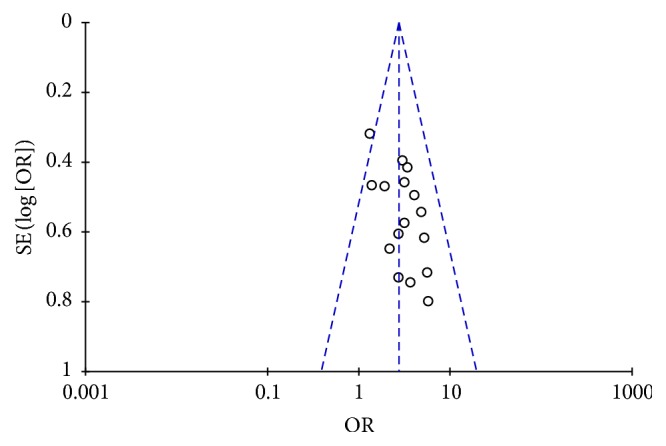
The funnel plot of the overall response rate of Jade Screen combined with desloratadine versus desloratadine alone for the treatment of chronic urticaria.

For the 16 research studies included, a funnel plot of overall response rate was drawn with the OR value of each paper as the *x*-coordinate and standard error logOR value as the *y*-coordinate. The analysis result of the funnel plot showed no mean symmetric distribution on the two sides of the plot, suggesting some publication bias possibly due to the limited number of the published papers with negative results, Figure 9.

## 4. Discussion

Chronic urticaria is a clinically common skin disease that is hard to heal and often reoccurs even after it is suppressed; thus, it is worrisome to patients. The cause of this disease might be associated with food, drug, infection, inhalant, physical, genetic, endocrine, and mental factors [32]. However, it is difficult to identify the real etiological and predisposing factors in most patients, and relapse cannot be absolutely avoided even if a possible etiological factor is determined. The current treatment is mainly to use Western medicine to control the symptoms, but its effect is very limited. Thus, it is imperative to identify a novel therapeutic regime. With traditional Chinese medicine as the guideline, the present research found and verified a classic prescription with fewer side effects and a low reoccurrence rate, which could be a new method of treating chronic urticaria through the combination of Chinese traditional and Western medicines.

IgE is primarily generated from the plasma cells in the mucous membranes of the respiratory tract (nasopharynx, tonsils, and bronchi) and the digestive tract. It plays a crucial role in mediating the occurrence and development of allergic diseases [33]. Modern pharmacological research [34] suggests that by tonifying qi and strengthening exterior, Yupingfegn can boost immunity, improve allergic constitution, inhibit mastocyte release of bioactive substances, strengthen the T cell-mediated cellular immunity effect, increase the number of T cells, and improve lymphocyte transformation rate [35]. Therefore, Jade Screen powder has been widely used to treat allergic skin diseases. Desloratadine has a long action time, a stronger antihistamine effect than other drugs of the same kind, a better antiallergic effect, selective cholinolytic activity, difficulty in passing through the blood-brain barrier, no central sedation and good patient compliance [36]; for these reasons, it has been widely used in the clinic as a first-line drug and a conventional antihistamine treatment. However, this drug only targets the disease process, so it cannot effectively control relapse. For the treatment of chronic urticaria, Jade Screen combined with desloratadine makes full use of Jade Screen to comprehensively strengthen the body's immunity, play the role of two-way regulation, improve IgA in the mucosal immune system, and act together with desloratadine to reduce IgE in hypersensitivity, regulate IgE-mediated humoral immunity, and significantly lower the IgE level of patients with urticaria [37].

In this research, we made a systematic evaluation on the functions of Jade Screen combined with desloratadine in treating chronic urticaria. Through a meta-analysis of the published studies, this research increased the sample size, enhanced the research credibility, and provided a reliable theoretical basis for clinical use.

First, the results of total effective rate and recurrence rate suggested that the total effective rate of the combination of traditional Chinese and Western medicine in treating chronic urticaria was higher than the single use of desloratadine, while the recurrence rate was lower than the single use of desloratadine.

Second, the results of occurrence rate of adverse reaction revealed that the combination of traditional Chinese and Western medicine and the single use of desloratadine showed no difference in the occurrence rate of adverse reactions. The adverse reactions of both treatments were mainly characterized by xerostomia, nausea, headache, dizziness, drowsiness, and, to a lesser extent, lack of strength.

Third, the results of the symptom score and total serum IgE showed high heterogeneity. The primary reason could be the small number of articles reviewed and their poor quality. Second, the published research literature about the curative effect of chronic urticaria in the clinic, the criteria for clinical symptom assessment, and intervention measures performance still cause much confusion, and no unified cure-judging criteria have been adopted, thus increasing the heterogeneity between the results of different research and having a high risk of selective bias.

The results of this study have satisfied our goal of this meta-analysis. The result has proven the effective therapeutic effect, fewer adverse reactions, and good safety of the traditional Chinese prescription Jade Screen in treating chronic urticaria. Thus, it can be used as an option for the treatment of chronic urticaria.

## 5. Limitations and Advantages

This research assesses the curative effect of Jade Screen in treating chronic urticaria according to the Methods for Cochrane Systematic Review for the first time. With limited cases included and low quality, the research fails to make a completely reliable positive conclusion. The limitations of the research included are as follows: (1) the research sites of all the articles were located in China, which is very likely to cause potential location bias; (2) all patients included were Chinese, and all the articles were written in Chinese or English, possibly resulting in potential selection bias and English language bias. (3) The articles included varied in dose, which may lead to potential drug dose bias. The advantages and deficiencies of the research were as follows. Deficiencies, (4) the methodological quality of the research included was low. All the research included mention “randomization”, but only a few expound on how to generate a random sequence, and most research failed to describe how to realize allocation concealment. (5) The articles included have no unified diagnosis and efficacy determination criteria, increasing the heterogeneity between the results of different research and a high risk of selective bias. On the one hand, there is absence of the latest guidelines [38, 39] prepared by evidence-based medicine methods in China; on the other hand, Chinese scholars probably do not pay much attention to it. Based on the above-mentioned bias factors, the efficacy and safety assessments of Jade Screen combined with desloratadine in treating chronic urticaria still require high-quality clinical randomized controlled trials. It is necessary to conduct large sample, multicenter, high-quality clinical randomized controlled trials with long-term follow-up in the future.

With the development of examining approaches and the emphasis on patients' quality of life in recent years, many articles have included laboratory indices and quality of life into the clinical efficacy system of chronic urticaria [40]. For example, drug therapeutic effect and safety assessments are carried out based on changes in laboratory indices such as the Q-T segment in the ECG, blood routine tests, creatinine clearance rate, IgE and plasma histaminase before and after treatment [41, 42]. With the furthering of traditional Chinese medicine in exploring the mechanism of chronic urticaria, there is an urgent need for China to establish an objective, effective, and extensive evaluation system that complies with the theory of TCM in order to better meet international standards. In future studies, we suggest using the Urticaria Activity Score (UAS) and quality of life to perform therapeutic evaluation and provide complete original data to further improve this meta-analysis in the future.

## 6. Conclusion

(1) The total effective rate of the combined use of Jade Screen and desloratadine was greater than that of desloratadine alone.

(2) The recurrence rate of the combined use of Jade Screen and desloratadine was greater than that of desloratadine alone.

(3) There was no difference in the incidence of adverse reactions between the two treatments.

(4) The present research results have showed that, in the treatment of chronic urticaria, the combination of traditional Chinese medicine and Western medicine is significantly better than using Western medicine alone. The therapeutic method provides a new thought for the treatment of chronic urticaria. However, due to the low quality of the currently selected articles, it is a necessity to carry out randomized controlled trials with strict designs in the future.

## Figures and Tables

**Table 1:** The basic characteristics of the 16 RCT studies.

Author/year	Groups	Sample size	Age (median or mean or range)	Course of disease	Course of treatment (days)	Intervention	Outcomes
Zhang et al., 2008 [16]	EP	50	19–60	21.3 months	28	(1)+(2)	①②③
	CG	50	19–60	22.5 months	28	(1)	
Jiang et al., 2009 [17]	EP	32	18–52	6–24 weeks	84	(1)+(2)	①②④
	CG	30	18–50	6–25 weeks	84	(1)	
Hu et al., 2011 [18]	EP	28	21–58	9 weeks–11 months	28	(1)+(2)	①
	CG	26	21–58	9 weeks–11 months	28	(1)	
Cai, 2012 [19]	EP	62	18–75	2 months–10 years	40	(1)+(2)	①②③④
	CG	58	18–75	2 months–10 years	40	(1)	
Liang, 2012 [20]	EP	60	34 ± 8.9	1.1 ± 0.25 years	28	(3)+(4)	①②③
	CG	60	36 ± 9.7	1.6 ± 0.5 years	28	(3)	
Qu, 2013 [21]	EP	43	12–45	6 weeks–2 years	28	(1)+(2)	①②
	CG	43	13–43	6 weeks–2 years	28	(1)	
Su et al., 2013 [22]	EP	59	12–60	≥6 weeks	28	(3)+(4)	①②③
	CG	58	12–60	≥6 weeks	28	(3)	
Wang, 2013 [23]	EP	99	35–60	3 months–12 years	28	(1)+(2)	①②③
	CG	82	35–60	3 months–12 years	28	(1)	
Wei and Zhang, 2013 [24]	EP	41	34.5 ± 10.7	≥6 weeks	28	(3)+(2)	①②③
	CG	40	37.2 ± 12.9	≥6 weeks	28	(3)	
Zhu, 2013 [25]	EP	36	34.60 ± 4.20	2 months–3 years	21	(1)+(2)	①②③
	CG	28	34.60 ± 4.20	2 months–3 years	21	(1)	
Xie, 2014 [26]	EP	60	16–58	2 months–8 years	56	(1)+(2)	①②
	CG	60	16–58	2 months–8 years	56	(1)	
Fu et al., 2015 [27]	EP	100	35.0 ± 3.0	4.1 ± 1.6 years	56	(3)+(4)	①②③④⑤
	CG	110	36.5 ± 2.5	4.4 ± 1.3 years	56	(3)	
Li, 2015 [28]	EP	62	18–56	2 months–3 years	28	(1)+(2)	①②
	CG	48	20–58	2 months–4 years	28	(1)	
Tian and Huang, 2015 [29]	EP	86	18–62	8 weeks–5 years	28	(3)+(4)	①②③⑤
	CG	86	18–63	9 weeks–7 years	28	(3)	
Qin et al., 2016 [30]	EP	48	37.2 ± 9.5	1.0 ± 0.4 years	28	(3)+(4)	①②③
	CG	48	36.7 ± 8.6	1.2 ± 0.5 years	28	(3)	
Zhao, 2016 [31]	EP	35	39.25 ± 6.23	4.49 ± 2.26 years	30	(1)+(2)	①②③④
	CG	35	38.28 ± 7.23	3.65 ± 2.11 years	30	(1)	

(1) Desloratadine: 5 mg po qd; (2) Jade Screen: po tid + Desloratadine: 5 mg po qd; (3) desloratadine: 8.8 mg po qd; (4) Jade Screen: po tid + desloratadine: 8.8 mg po qd; EP: experiment group; CG: control group; ① total effective rate; ② adverse reaction; ③ recurrence rate; ④ symptom score; ⑤ serum IgE level.
